# Climate change and health in Israel: adaptation policies for extreme weather events

**DOI:** 10.1186/2045-4015-2-23

**Published:** 2013-06-27

**Authors:** Manfred S Green, Noemie Groag Pri-or, Guedi Capeluto, Yoram Epstein, Shlomit Paz

**Affiliations:** 1 School of Public Health, University of Haifa, Haifa, Israel; 2 Faculty of Architecture and Town Planning, Technion, Israel Institute of Technology, Technion, Israel; 3 Department of Physiology, Faculty of Medicine, Tel Aviv University, Tel Aviv, Israel; 4 Department of Geography and Environmental Studies, University of Haifa, Haifa, Israel

## Abstract

Climatic changes have increased the world-wide frequency of extreme weather events such as heat waves, cold spells, floods, storms and droughts. These extreme events potentially affect the health status of millions of people, increasing disease and death. Since mitigation of climate change is a long and complex process, emphasis has recently been placed on the measures required for adaptation. Although the principles underlying these measures are universal, preparedness plans and policies need to be tailored to local conditions. In this paper, we conducted a review of the literature on the possible health consequences of extreme weather events in Israel, where the conditions are characteristic of the Mediterranean region. Strong evidence indicates that the frequency and duration of several types of extreme weather events are increasing in the Mediterranean Basin, including Israel. We examined the public health policy implications for adaptation to climate change in the region, and proposed public health adaptation policy options. Preparedness for the public health impact of increased extreme weather events is still relatively limited and clear public health policies are urgently needed. These include improved early warning and monitoring systems, preparedness of the health system, educational programs and the living environment. Regional collaboration should be a priority.

## Background

Human-induced emission of greenhouse gases into the lower atmosphere is increasingly contributing to the warming of the earth's surface. There is now strong evidence for recent climatic changes. There have been significant temperature increase, more extreme weather events, sea level rise and shrinkage of most glaciers and snow cover [1,2]. According to the UNEP, climate change "is the major, overriding environmental issue of our time, and the single greatest challenge facing environmental regulators. It is a growing crisis, and has economic, health and safety, food production, security, and other dimensions" [3]. One of the major concerns for climate change is its impact on human health.

In 2007, the Intergovernmental Program on Climate Change (IPCC) produced the fourth report on the potential health effects of climate change [4]. The findings were summarized as follows: "Projected climate change-related exposures are likely to affect the health status of millions of people, particularly those with low adaptive capacity, through increases in malnutrition and consequent disorders, with implications for child growth and development, increased deaths, disease and injury due to heat waves, floods, storms, fires and droughts, an increased burden of diarrheal disease, an increased frequency of cardio-respiratory diseases due to higher concentrations of ground-level ozone related to climate change and an altered spatial distribution of some infectious disease vectors." Moreover, in a recent report, the IPCC (2012) stressed again that there will be an increase in the severity of adverse public health impacts in climate-related disasters such as storms, floods, heat waves, drought and wildfire [5].

International efforts are being made to mitigate the effects of climate change by reducing the production of greenhouse gases. Despite these efforts, it is clear that climate change is already happening and cannot be prevented. Thus, comprehensive plans are necessary to ensure effective adaptation to different scenarios. The term "adaptation" refers to the process of adjustment to actual or expected climate and its effects in human systems, to moderate harm or exploit beneficial opportunities [5].

Due to the many uncertainties, a so-called "low-regrets" approach has been proposed. Low-regrets measures provide benefits under current climate conditions and a range of future climate change scenarios. They are useful and readily available starting points for addressing projected trends in exposure, vulnerability and climate extremes. They have the potential to offer benefits now and lay the foundation for addressing projected changes [5]. These measures are based on directing resources of preparedness, which will have a positive impact on public health, regardless of the impact of climate change. In addition to recommending specific preparedness measures, The World Health Organization (WHO) has called for "strengthening public health systems, emergency response programs, and research around the globe" [6]. Although general guidelines for adaptation to climate change exist, preparedness plans should be tailored to the specific conditions and needs of the various regions of the world.

### Climate change in the eastern mediterranean basin

Since the 1960s, the Mediterranean region has become warmer with a significant increase in the frequency, intensity and duration of heat waves [7]. In addition, the basin is characterized by a reduction in the potable water availability as a result of decrease in the total amount of precipitation, change in rainfall patterns [5,8], and water overuse by the growing population. In the Mediterranean basin, mutual enhancement (positive feedback) between the two conditions, droughts and heat waves, has been identified [5].

All of these trends have been detected in the Eastern Mediterranean Basin in general, and most, if not all, have been detected in Israel. According to the IPCC (2012), an increase in warm days and nights has been observed, in parallel with a reduction in cold days and nights. During the last few decades, the summers in Israel have become significantly warmer [9], with an increase in the frequency and the severity of heat waves [10,11]. The major contributor to the rise in the mean temperature is the significant increase in the minimum temperature [9].

Although Israel's climatological community agrees that the region is experiencing a warming trend [12], less consensus has been reached regarding the change in rainfall patterns. In 2002, Alpert et al. pointed to the paradoxical increase in Mediterranean rainfall intensity despite the decrease in total amounts [8]. However, in 2011, the Israel Meteorological Society did not identify a significant decrease in rainfall amounts in Israel, nor in their seasonal distribution [9]. In a recent paper, Ziv and Saaroni showed an increase in the relative humidity, particularly along the Israeli coastline [13].

The Israel Climate Change Information Center (ICCIC) report (2011) [12] summarized the main predicted impacts of climatic changes on the country: A decadal increase in average annual temperature, an increase in the duration and intensity of heat waves, a reduction in the average quantity of precipitation, an increase in desertification processes in southern Israel, increased risk of floods, increased probability of forest fires and changes in sea level.

In this paper, we review the potential health consequences of extreme weather events resulting from climate change in Israel as a case study of the Mediterranean region, and propose adaptation measures that need to be incorporated in public health policy.

## Extreme weather events and their impacts on human health, with particular attention to Israel

The consequences of extreme weather on human health can be divided into two categories; direct and indirect. Direct impacts are deaths, disease and injury due to heat waves, floods and storms (including the risks from sewage and chemical contamination) and mental stress, poor air quality, increased pollen, reduced food safety due to higher temperatures and greater exposure to ultraviolet radiation. The indirect effects are widening health and social inequality due to rising global food prices, and food insecurity, causing population displacement and migrations. In the Eastern Mediterranean region, the most common extreme weather events are heat waves.

### Heat conditions

The National Oceanic and Atmospheric Administration (NOAA) (2009) defines a heat wave as: "a period of abnormally and uncomfortably hot and unusually humid weather…typically, a heat wave lasts two or more days" [14]. In fact, it is difficult to establish a global definition because of the geographically variable nature and impact of heat waves [15], which depend on the local climate of each region together with the level of the population’s acclimatization.

Heat-related illnesses (e.g., heat cramps, heat exhaustion, heat syncope, heatstroke or heat rashes) can occur when high ambient temperatures overcome the body's natural ability to dissipate heat. Older adults, young children and persons with chronic medical conditions are particularly susceptible to these illnesses and are at high risk for heat-related mortality [16]. Heat stress can occur when both air temperature and humidity are high [17]. Here again, it depends on the local climate and the public acclimation. See, for example, the U.S. heat stress index, NOAA (2013) [18].

The health impact of the increasing frequency of heat waves is already being felt in countries such as Germany [19], China [20], Russia [21], the United States [22,23], Australia [24] and Korea [25]. It has been estimated that the heat wave in Europe in 2003 resulted in more than 70,000 deaths [26-32]. These were mainly older persons living independently in the community, and people with chronic cardiovascular and respiratory disease [33-40]. Several studies carried out in recent years in different countries suggested that the short-term risk of myocardial infarction and other cardiovascular diseases increases during days of extreme hot weather, which are becoming more frequent as a result of climate change [41-43]. Other health effects of heat waves might include exacerbation of psychiatric conditions and respiratory illness [44].

No studies in Israel have reported an increase in cardiovascular disease during heat waves. However during the summer months, more hospital admissions due to cardiovascular disease were reported during warmer days than colder days [45]. In one study on the effects of heat on the number of emergency room (ER) visits in Israel, the contribution of mean daily temperature to the number of ER visits was small but significant [46]. The authors reported that the number of visits to ERs increased by 1.47% per 1°C increment in ambient temperature. Nevertheless, in the same study, the effect of humidity on the number of ER visits during heat waves in Israel was found to be negligible.

The lack of reports of increased cardiovascular morbidity during heat waves in Israel may represent the reality or, alternatively, may be a result of the incomplete analyses of the available data. Excess mortality from heat waves may have occurred, but has not been documented. This may be due to the relatively small size of the population, where small-scale effects are difficult to detect, or to inadequate analysis of available data. Furthermore, the lack of reporting on the health impacts of heat waves in Israel may be due to the structure of the monitoring system of deaths and causes of death, which may lack the sensitivity required to detect the direct effects of heat waves on mortality. It is also possible that behavioral adaptation of the Israeli population has mitigated the health impact of heat waves.

Nevertheless, based on the experience in Europe in 2003, it is possible that Israel will face significant mortality as a result of a prolonged extreme heat wave in the future. Due to the Mediterranean climate, Israel’s existing infrastructures for dealing with heat waves, (e.g., air conditioning) are probably better than those that were available in Europe in 2003. Nonetheless, special attention to the preparedness for protecting vulnerable groups, including low socioeconomic persons, is still essential. Without proper adaptive planning that takes into consideration both the population growth and the possible increase in extreme heat waves in Israel, climate change will result in significant adverse health effects and additional costs for the Israeli public health sector in future decades [47].

#### Heat conditions and air pollution

Exposure to pollutants, such as airborne particulate matter, has been shown to be associated with a rise in hospital admissions because of respiratory diseases [48]. Interaction between air pollution and extreme heat during heat waves might further increase the incidence of these diseases [49,50]. For example, asthma attacks and other respiratory diseases are expected to increase during heat waves due to interactions with air pollution in general and from a rise in the frequency of wildfires [47,51]. Indeed, a recent analysis [52] found a strong positive association between the ambient daily temperatures and PM10 concentrations, and the number of patients arriving on the same day to the Rambam Medical Center (Israel), controlled for autoregressive effects, day of the week and season of the year.

#### Heat conditions and infectious diseases

In general, infectious diseases are not related to extreme heat events. However, in recent years, increasing attention has been paid to the linkage between extreme heat conditions and some *vector*-*borne* diseases. These include Dengue and West Nile virus [53-55]. In addition there have been outbreaks of food-borne diseases [56] due to inadequate cooling of food served in public places and spoiling of foodstuffs as a result of power failures caused by excessive energy demands. However, the current paper does not focus on possible associations between heat spells and outbreaks of infectious diseases since this is a wide and complex issue that justifies a separate review.

#### The impact of heat conditions on vulnerable groups

##### a. Elderly and the chronically ill

The elderly and the chronically ill are vulnerable groups at high risk of heat stress [44,57]. A number of studies around the world have shown that older persons and people with pre-existing chronic conditions (e.g., diabetes, cardiovascular diseases, respiratory diseases) have higher susceptibility to heat and are at high risk of increased morbidity during heat waves [39,57-60]. For example, in a study in Seoul [61], it was found that among elderly living in poor housing conditions, body temperature increased by 0.07°C with each 1°C increase in the mean outdoor temperature.

Excess mortality among the elderly during heat waves has been reported in Australia [62]. It has been predicted that, assuming no change in adaptation measures, the impact of projected high temperatures on annual mortality in the older age group, for capital cities only, may increase by a half to three times, depending on the greenhouse gas emissions scenario [62].

In Israel, the risk of heat waves is not dissimilar from the risk in Australia, which also has regions with Mediterranean climate type. Thus, without an adaptation plan that will take into account the expected increase in heat waves and the aging of the population, the predictions for Israel might well be similar to the predictions for Australia.

Among the elderly and the chronically ill in Israel, excess morbidity and mortality from heat stress might have occurred, but has not been documented. The actual monitoring of the condition of those groups is complex on account of the lack of an adequate monitoring system for heat-related morbidity and mortality and the failure to mention heat stress on death certificates. In addition, older and chronically ill people may be treated mainly in the community where the data from monitoring are often less specific. Thus, the lack of data might conceal the true extent of the effects of heat stress on morbidity and mortality among the elderly and the chronically ill. In Israel, these populations are included in the Ministry of Health risk groups to be monitored in cases of extreme weather events [63-65].

##### b. Poor and indigent people

Poor people often lack well-designed, adequately insulated housing and air conditioning. They will be more affected by heat waves if they lack the means to protect themselves [57,66]. No reports exist on the adverse health effects of extreme heat and heat waves on poor populations in general, and in Israel in particular. It is likely that, to save money, poorer people in Israel make less use of home cooling. Thus, it is possible that they are already suffering from increased morbidity during heat waves, but this goes unnoticed because of the limitations of the monitoring system.

##### c. People with mental illness and physical disability

Informal evidence shows that well-being, morale and mental health are affected by changes in the environment and the perceptions of it [62,67]. Heat waves were found to increase the risk of mortality also for people with mental illness by 4.9% for each 1°C increase in ambient temperature above the 93rd percentile of the annual temperature distribution [68]. Evidence suggests that patients with schizophrenia are less tolerant of heat [69,70].

In Israel, several studies have found a seasonal variation in the admission rates of patients with mental illness suffering from different conditions, as well as a linear correlation between admission rates and the mean maximal monthly temperature [70,71]. Even with the evidence mentioned above, it is difficult to determine the exact effect of heat waves on acute or long-term mental illness. This determination will require a much closer examination than is performed in Israel today, of the specific timing of mental illness episodes and the environmental conditions at the time.

In addition to the possible effects of heat waves on acute psychiatric episodes, extensive and frequent heat waves may also affect the health of people with special needs. For example, extreme heat could severely limit outdoor activities for people with serious physical disabilities due to the extra effort this requires.

##### d. Ethnic and cultural minorities

As far as we know, little research has been performed concerning the health effects of heat waves on the different minorities living in Israel. Different ethnic and cultural minority groups in Israel have traditional customs that may cause difficulties in coping with heat waves. For example, many Bedouin in the Negev desert live in tents and are poorly protected against heat waves. There is evidence of some behavioral adaptation which could limit the adverse effects of heat [72]. Heat waves might have already affected morbidity and mortality rates among minorities in Israel, but this has not been documented because they are not evaluated separately from the total population. As the frequency of heat waves increases, disease and death among minority groups in Israel may be proportionately greater than in the total population.

### Extreme cold spells

Although global temperatures are rising, extreme cold weather events are increasing in frequency [6]. Different studies conducted around the world have reported an increase in mortality during cold spells [73-75]. Most of the cardiovascular diseases show a seasonal pattern with elevated morbidity during the cold season, leading to hospitalizations on colder days [76-78]. Infections of the respiratory tract are more common during the winter months. Although Israel has not experienced a significant increase in the frequency of severe cold waves, it still important to prepare for this possibility as there is a non-negligible risk of such change in the future.

**Figure 1:**
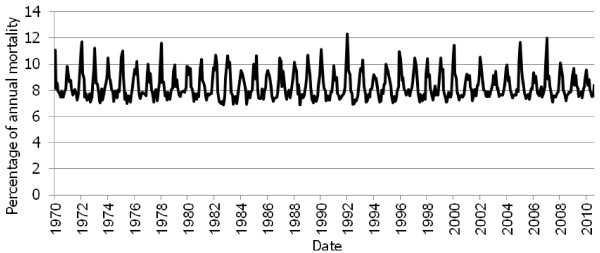
**Monthly Percentage of Annual Mortality, Israel: 1970–2010.** (Source: State of Israel, CBS, 2012. Data available at: http://www.cbs.gov.il/ts/databank/series_func_v1.html?level_1=2&level_2=2&level_3=1 Retrieved: 3.1.2012).

Overall mortality increases in winter in Israel (Figure 1), and cases of preventable cold-related illnesses occur resulting in hospitalization and death. For example, the total mortality rate from myocardial infarction in Israel is seasonal, with higher rates during the winter months and during colder years [79]. However the specific health impact of cold spells or more unusually extreme winter events have not been reported.

It is, of course, possible that generally warmer weather may reduce the extent of winter peaks in mortality in Israel. Since the climate is generally warm, the general population is often unaware of the potential dangers related to exposure to extremely cold weather, especially to newborns, premature babies and the elderly.

#### The impacts of cold spells on vulnerable groups

##### a. Elderly and the chronically ill

The elderly and the chronically ill are especially susceptible to the adverse effects of extreme cold, because of their lower resistance to illness as well as the chronic use of medications. A study in Moscow, which examined the health effects of two cold spells that occurred during 2006, found that their cumulative effects on mortality were significant only in the 75+ age group, in which 370 extra deaths occurred [75]. A study in the Netherlands found a V-shaped relationship between mortality and temperature. The researchers found that the optimum low temperature value was 16.5°C for people aged 65 years or above. Mortality increased by 1.37% for each degree decrease below the optimum. The average excess mortality during the cold spells was 12.8%, and was mostly attributable to the increase in cardiovascular mortality and mortality among older persons [80]. Although Israel has a warmer climate than the countries in the aforementioned studies, according to the Israel Ministry of Health, in the last few years, an increasing number of elderly people have suffered serious injuries as a result of cold exposure. Some injuries required hospitalization and were even the cause of death [81].

##### b. Children

Children represent a particularly vulnerable group that is likely to suffer disproportionately from both direct and indirect adverse health effects of extreme cold [82]. An increase in extreme cold spells will increase the risk of morbidity and mortality in the sensitive population of young infants [75,83]. It is possible that the rate of cold-related injuries in babies in Israel is greater than currently reported, and that the under-reporting is a result of the limited capabilities of the existing monitoring system of morbidity and mortality. Cold injuries to babies may increase, especially since the Israeli population may be less prepared to handle extreme cold.

##### c. The poor, indigent and people with special needs

Poor and indigent people are more susceptible to the effects of extreme cold, as they are less equipped to deal with them than people in higher socioeconomic strata. It might be expected that during extremely cold days and nights, poor and homeless people would seek shelter in public places such as hospitals. According to a study in the United Kingdom, attendance of homeless people in emergency rooms was more frequent during evenings and nights when the temperature dropped, and a relatively high proportion (17.4%) of them left without being seen by a doctor. The same research found a small positive correlation between daily attendances of homeless people in emergency rooms and minimum daily temperature [84]. Those with mental illness may be at increased risk of the adverse effects of extreme cold since some studies have found that the rates of suicide and depression tend to escalate during cold, rainy days [85].

### Natural disasters: fires, floods and droughts

Growing evidence shows that climate change is increasing the frequency and intensity of climate-related natural disasters [57,86]. The effects of global warming on rainfall, floods, drought, storms, fires and other natural phenomena have far-reaching public health effects not only on environmentally associated disease outbreaks but also on global food and water supplies and safety, population movements and maybe even death rates [47,51,58,87-90]. As a result, an adaptation plan must be established, including a combination of economic, environmental, legal, regulatory and primary public health measures [86]. Water-borne, food-borne and vector-borne diseases might increase during floods, or be enhanced by prolonged droughts [89,90]. The acute stress from natural disasters might increase the incidence of diseases such as myocardial infarction.

Droughts are likely to increase as a consequence of climate change [91]. According to the IPCC (2012), there is medium certainty that droughts will intensify in the 21st century in the Mediterranean Basin, due to reduced precipitation and/or increased evapotranspiration [5]. Drought might increase the probability of wildfires. Indeed, during the past several decades, a sharp increase in fire events in the Mediterranean forests has been observed [92], such as the severe wildfire in December 2010 in the Carmel Forest, Israel [93]. This was the largest forest fire in the history of Israel, which exposed between 4,000 and 7,500 hectare to the worst fire ever recorded [94]. The weather conditions prior to and during its occurrence were exceptional. Summer 2010 was the warmest on record and the following fall was the warmest and driest in the previous 40 years with a precipitation amount of approximately 10% of the perennial average rate of the season. As a result, the vegetation was unusually dry for the time of year. During the days of the wildfire, the air temperature was very high and the relative humidity was extremely low, below 10%. These conditions, together with strong, dry easterly winds, resulted in the rapid spread of the fire [11,93].

Recurrent floods occur in Israel, mostly in the arid southern regions. During January 2013, severe floods occurred in many areas of the country as a result of extreme storm conditions. In fact many hydrological systems reported the highest ever recorded water flows [95]. This type of flooding causes damage to property and drowning. Thus, it is quite possible that the occasional floods and/or the ongoing drought may have had adverse impacts on health that have not been documented (in particular due to the increasing frequency of droughts in Israel). This phenomenon, if prolonged, may cause salination and contamination of ground water, affecting agriculture and damaging public health.

#### The impacts of natural disasters on vulnerable groups

The acute stress caused by natural disasters might adversely affect the elderly and chronically ill populations, who have fewer coping mechanisms and are highly sensitive to the health effects of stress. Children are at highest risk of malnutrition, with long-term implications for their overall development [57,58]. Extreme weather events may increase the risk of long-term mental health issues such as posttraumatic stress disorders [96], anxiety and depression [47,51]

## Health policy recommendations for adaptation to extreme events due to climate change

The health dimension of vulnerability to extreme events includes differential physical, physiological and mental health effects in different regions and on different social groups. It also includes the possible impact of extreme weather events on the provision of health services (e.g., infrastructure and facilities). Vulnerability can also be understood in terms of functionality related to communication, medical care, maintaining independence, supervision and transportation [5]. The public health policies for adaptation to extreme climate events essentially require sound and effective preparedness for response to heat waves, cold spells, floods and droughts.

In particular, health policies that address the special needs of vulnerable groups must be identified, and where necessary, given special attention. In general, policy recommendations should address at least five areas: early detection of extreme weather events, preparedness of the health systems, monitoring of morbidity and mortality, public education and the living environment. These recommendations are grounded in the review of the literature presented in previous sections of this paper, along with our own insights into Israeli society and its policy development processes. In light of the space constraints of a journal article, these recommendations are perforce presented in general terms and could easily be elaborated in appropriate forums.

### Early detection of extreme weather events

The meteorological services are responsible for the updated alerts on the possibility of extreme weather events. Thus, a prerequisite for any monitoring system for the early detection of extreme weather events is productive collaboration between the meteorological service and the health authorities. An action plan should be developed, containing definitions of "changes in the level of alertness and action" for the preparedness for heat waves and cold spells. Exercises on an annual basis should be carried out to evaluate the functioning and quality of the alert system.

### Preparedness of the health system

Policy recommendations should address the emergency services’ delivery, governance and regulations, the health workforce, medical products, financing and assisting other countries in addressing the health effects of climate change. Special attention should be given to people with limited ability and who are unable to leave their homes independently. During heat waves and cold spells, all individuals in the high risk groups should be contacted personally by employees of the Ministry of Welfare, either by telephone or a home visit. In case of need, those individuals should be evacuated to hospitals or to air-conditioned public places. This should be done in addition to, and not instead of, the current recommendation that family members should visit individuals in risk groups, such as older persons, on hot days.

Moreover, the Israel Ministry of Health defined heat wave as sequentially three days or more with at least 32.2°C. According to their report, extreme heat wave occurs when the temperature is 30°C and the relative humidity is at least 70%, or when the relative humidity is lower but the temperatures are higher [64]. Since these conditions exist in many parts of the country along the whole summer, we call for a more relevant new definition.

### Monitoring

**Figure 2:**
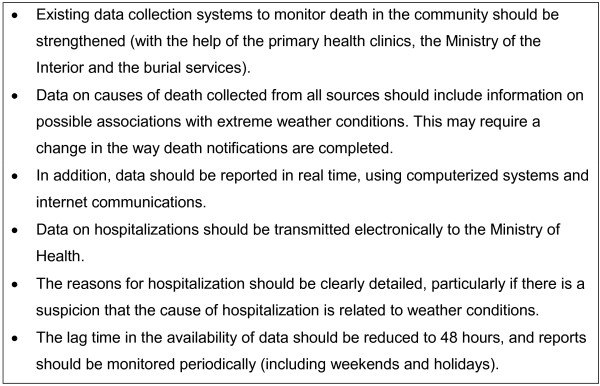
Recommended data needs for preparedness for extreme weather events.

**Figure 3:**
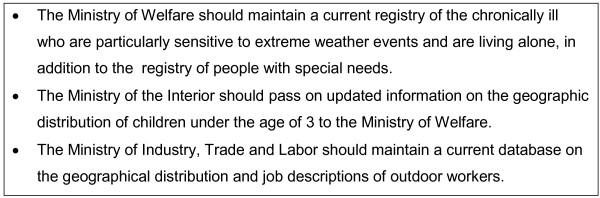
Recommended registries of the demographic details and geographic location of vulnerable groups and individuals that should be maintained and updated regularly (older persons, children, chronically ill, people with special needs, outdoor workers).

There is a need for a monitoring system for the health effects of climate change, including indices of total and cause-specific morbidity and mortality. As soon as a heat/cold wave begins, causes of morbidity and mortality should be reported to a central department in the Ministry of Health within 48 hours (Figure 2). The data should be analyzed and reviewed on a daily basis. This applies to both hospitals and community health clinics. Registers of the demographic details and geographic location of vulnerable groups and individuals should be maintained and updated regularly; (elderly, children, chronically ill, people with special needs, outdoor workers) (Figure 3). At the same time, it should be kept in mind that the experience in other countries suggests that the development of such registries can be challenging, but that these challenges can be overcome with adequate resources and ingenuity.

### Education

There is a need for improved educational programs for the public and for health professionals on how to adapt to the changes in the climate. Guidelines for behavior in case of heat waves and cold spells for the general public, as well as guidelines for the proper treatment of heat and cold injuries in hospitals, have been published by the Israel Ministry of Health [63,64,97]. These materials should be updated regularly and made more accessible to the public. They should be displayed clearly in emergency rooms and primary care clinics. In addition, these guidelines should be included in the teaching curriculum for medical students and other health professionals. No guidelines for public workers have been published regarding the identification of the onset of heat-related illness and the guidelines for prevention. Such guidelines should be developed and displayed.

### The living environment

The potential impact of climate change requires a conceptual adjustment in the way buildings and cities can be adapted (shade, wind exposure, thermal comfort, adjustment to extreme events such as floods and sea level rise, etc.). Currently, health aspects are not given sufficient consideration in existing compulsory building regulations. It is important that the building codes will require new buildings to be designed to comply with green standards [98] considering design strategies, such as improved insulation, window shading, thermal mass and natural ventilation. This is not only for mitigation and energy saving, but also to achieve indoor air quality and thermal comfort throughout the year and to ensure that they remain relatively cool in case of a heat wave. In addition, there is a need to reduce dependence on air conditioning (through shading and natural ventilation) which makes high and increasingly unsustainable demands on energy.

Special consideration should be given to existing buildings, offering incentives to perform green retrofit and to adapt them to updated green standards, improving the building envelope to reduce energy consumption and improve thermal comfort. The use of energy-efficient heating, ventilation and air-conditioning (HVAC) systems to achieve thermal comfort during extreme events should be encouraged.

Open areas, parks and streets can provide a vital space for public urban life throughout the entire year. The public’s successful use and enjoyment of this space depends heavily on microclimatic conditions that affect thermal comfort. Appropriate comprehensive planning should consider ways of bringing together the different urban components to make use of renewable energy sources for passive heating and cooling of buildings, to reduce the heat island effect (the built-up areas that are hotter than nearby rural areas) and to create open spaces that can provide enjoyment and sustain a healthy urban lifestyle.

The Israeli government has started taking steps toward the achievement of this goal, but has reduced its investment in the plan due to budgetary considerations [99]. This plan should be updated and reactivated as it is likely to enhance both mitigation and adaptation efforts for climate change and to reduce the severity of its predicted health effects. Shelters should be continually maintained and improved so that they can be used for homeless people in case of a heat wave. Individuals from risk groups should be housed in buildings that are well-designed, properly insulated and equipped with air conditioning. All public spaces should be designed in accordance with green standards, including those proposed by the Israel Green Building Council, and should ensure the thermal comfort of users. Shading and ventilation should be provided in open spaces, particularly during the hot season [100].

## Conclusions

Based on predictive modeling, the average temperature might increase globally by between 2-5°C during the 21^st^ century [101]. Indeed, the impact of climate change is likely to worsen markedly in the coming decades in many parts of the world including Israel. Several climate change adaptation policies should be developed and tailored to the needs of Israel and other countries in the Mediterranean Basin. They should be directed particularly at reducing the adverse health effects resulting from the increase in extreme weather events due to climate change. These include the development of climate alert systems, improved preparedness of the health systems, enhanced monitoring of morbidity and mortality, education programs for both professionals and the public and management of the urban environment. In light of the possible interactive effect between air pollution and extreme weather events, efforts should be made to reduce air pollution to the minimum. The adaptation plans must take into account the special needs and cultures of minority groups, vulnerable groups, the elderly and the chronically ill. Efforts should be made to increase regional collaboration on these issues, by pooling knowledge, coordinating monitoring and alert systems and developing common adaptation policies relevant to the Mediterranean basin.

## Competing interests

The authors declare that they have no competing interests.

## Authors’ contribution

MG conceptualized the idea of the manuscript and was involved in all steps of the writing of the paper. SP was involved in all aspects of the writing of the paper including its conceptualization. NGP was involved in data collection and analysis and assisted with writing the article. GC assisted with writing the section of the article concerning the living environment. YE assisted with writing the section of the article concerning heat conditions. All authors helped in reviewing drafts of the article. All authors read and approved the final manuscript.

## Authors’ information

Manfred Green MD,PhD is an epidemiologist and professor and head of the School of Public Health at the University of Haifa. Until 2008, he was director of the Israel Center for Disease Control and a professor in the Sackler Faculty of Medicine at Tel Aviv University. He is board certified in public health, health administration and occupational medicine.

Noemie Groag Pri-or is a research assistant at the School of Public Health, University of Haifa. She has a MPH degree in health promotion. Her main interest lies in understanding the effects of environmental and policy factors on the health of the population as a whole and the health of women and children in particular. Guedi Capeluto DSc is an architect and Associate Professor at the Faculty of Architecture and Town Planning, Technion, Israel Institute of Technology. His research is focused on sustainable design, green architecture, intelligent buildings, daylighting, daylight access and solar rights in urban design. Yoram Epstein PhD, FACSM specializes in environmental physiology and ergonomics, with special interest in thermoregulation. Presently, an associate professor of environmental physiology in the Sackler Faculty of Medicine, Tel Aviv University and a senior investigator and consultant at the Heller Institute of Medical Research in the Sheba Medical Center, Tel Hashomer. Shlomit Paz PhD is a senior lecturer at the Department of Geography and Environmental Studies, University of Haifa. She is a climatologist, who investigates the impacts of climate change on the public health, with a main focus on the effects of the global warming on vector-borne diseases.
